# Odontological Society of Pennsylvania

**Published:** 1894-12

**Authors:** 

**Affiliations:** Odontological Society of Pennsylvania


### ODONTOLOGICAL SOCIETY OF PENNSYLVANIA.

At a regular meeting of this Society, held October 13, 1894, President Peirce in the chair, Dr. Wm. L. Fish, of Newark, N. J., read his paper on "Cervical Cavities and their Treatment."

(For Dr. Fish's paper, see page 758.)

### DISCUSSION.

*Dr. Truman.*—The clamp seems to be an effective one; but it is highly complicated. There are few clamps so well made that they are not liable to cause disturbance. I think those who are here will bear me out in the assertion that if a clamp be forced upon the gum margin, as that appears to be, irritation will necessarily follow; pathogenic germs will gather around the tooth that may eventually result in pyorrhœa alveolaris, or at least in inflammation of the pericementum.

This is not mere theory, for I have observed such complications for years, ever since the introduction of clamps.

The gentleman has asserted that retaining-points are a snare. Now, I am happy to say that I do not agree with him. Retaining-points were introduced, in the earlier days of the profession, in a form, perhaps, that was not satisfactory. I remember, twenty-five years ago, I called attention to retaining-points properly made as I considered they should be. I practised and taught for many years that mode of filling teeth and anchoring them. And I hold to-day that it cannot be relinquished except in special cases. What do you get by undercutting? If there is any break or any pressure you destroy the anchorages at once, and the filling is lost. If you want to hold a filling in a tooth you must anchor it somewhere, and I deny, positively, that retaining-points cause thermal action upon the nerve if they be properly placed, any more than deep undercutting or the filling itself, if it approximates nearly to the pulp.

*Dr. Broomall.*—I entirely agree with the essayist on the subject of retaining-points. It has always been my opinion that they are valuable simply as a good beginning on which to place a filling, and do not deem them useful for holding a finished plug in place. As the operator approaches the surface of the cavity, there is a tendency for such anchorage to break away. I myself always use undercuts, and I think them far preferable to retaining-points.

Dr. Truman then asked those opposed to retaining-points what they would do with a tooth that had no cavity in it that might be necessary to build out. "Formerly before we became so æsthetic that we would not have gold in front of the mouth, it became necessary to occasionally build down an incisor. Even now that happens, and what can you do with it unless you make retaining-pits? When teeth are perfectly smooth, I build these down by making a circle of pits all around the tooth-margin. Then placing a screw here and there, I insert the gold, and such fillings have remained secure and firm for years. I would like to ask the gentlemen who are opposed to this mode of filling how they would treat such a place or cavity."

*Dr. Broomall.*—Dr. Truman says he occasional puts in a screw, and I think he answers himself; whereas, if you have the retaining-points you run a risk of not getting firm union.

*Dr. Head.*—Does the gentleman mean to say that a screw fastened into the tooth-structure will have a more intimate connection with the tooth-structure than gold hammered in there.

*Dr. Broomall.*—No, I do not mean that. I contend that gold in the shape of wire screws is stronger than any you can put with a plugger into a small cavity that has just a retaining-point.

*Dr. Boice.*—I can heartily agree with Dr. Truman as to the influence of clamps on the teeth. I see it every day, and I have been thinking, for the last five years, that it would be better if we had never seen a clamp. I am using them less and less every day. The clamp presented here to-night is a very ingenious thing, and contains many valuable points we can utilize.

In reference to retaining-points, there is a light-house on the British coast that was fastened year after year with bolts into the rock, and every time the storms came, it would be washed away The bolts represent the same thing you have in filling teeth. It was my pleasure when I began to study dentistry, to come in contact with a man by the name of Head, and he said retaining-points were bad, therefore he wanted grooves. Now the light-house went away year after year, until a certain engineer cut grooves in the rock and put in the blocks, and then a key or centre block in that, and it has been standing for a hundred years. If you use that same principle in filling a cavity you have an easier filling and a better anchorage.

*Dr. Faught.*—I have listened with great pleasure to the paper. I am one of those who take the author's position in regard to retaining-points. I have observed numberless times, in cavities where fillings have been dislodged, that you will often find a little bit of gold in the retaining pit. This fact shows that when the filling is completed the retaining-point is of no further use, no matter how firmly it be condensed. I therefore shape the bottom of the cavity so that it will hold the gold without any necessity for such aid.

In regard to the clamp, I cannot believe it is such a complicated structure. It seems to be made according to true mechanical principles. Dental practitioners will use clamps, and if one uses a clamp at all, I believe this one will do the work better than any of those furnished to us by the depots.

*Dr. Fish.*—I take issue with Dr. Truman in reference to retaining-points. If a tooth is strong enough to allow an undercut, no matter how slight, if the walls of the cavity are made absolutely parallel, and that cavity is filled with perfect edges, the fillings will not come out.

The subject was then passed, after extending a vote of thanks to the essayist.

The President then called upon Dr. Brubaker to read his paper on "The Causation of Dental Erosion."

(For Dr. Brubaker's paper, see page 739.)

At the close of the paper, Dr. Brubaker exhibited a small chloride-of-silver battery, similar to those employed for generating electricity, and in removing superfluous hairs, and explained the manner of using it. By introducing the point of the needle into a hair-follicle, the current being applied, the bulb was destroyed. This, the doctor did as illustrating the manner in which the diseased glands might be eradicated.

While reading his paper, the essayist passed around for inspection some teeth with which he had experimented.

Dr. Peirce stated that he had examined the teeth before they were treated, and they were perfect, entirely free from any abrasion or cut.

Dr. Peirce asked Dr. Truman to open the discussion, and asked if the speaker's remarks were consistent with his experience.

*Dr. Truman.*—It confirms my own view of the matter. I never could credit the uric-acid theory, and I am confident that what Dr. Brubaker has said in his paper is absolutely correct.

I am also pleased to note that we have some ideas in regard to what may possibly be one of the causes of erosion. I think the examination of the teeth indicates, as he has stated, that a plan of investigation might be satisfactorily carried out that would lead to some results. I don't see, however, that his plan of destroying the glands will help matters very much. If the glands in the lip are destroyed, what will be the effect on the secretion necessary for the mucous membrane of the lip? I would like to ask Dr. Brubaker to answer that question. The lip would become very dry and difficult to manage. It is difficult now, for some of us to talk in our lectures; but aside from that, I question very much whether the glands are really the cause of erosion, although his research is an indication of what may occur.

Dr. Brubaker has very kindly alluded to some work I performed in this direction many years ago, while endeavoring to discover the cause of erosion. I carried the experiments into the night season, satisfied that the day hours gave a neutral reaction, and that it was impossible to secure satisfactory results during the period of the greatest flow of saliva. And I think I proved conclusively, certainly to my own mind, and to the minds of some others, that fermentation progressed through the night, and that it was not possible in the daytime. Dr. Kirk's idea was different, and he experimented on the glands of the lip in the daytime, and demonstrated by the aid of litmus-paper that there was an acid passing from the glands at all times during the twenty-four hours.

Therefore, we may conclude that at night the acid would not be more effectual in destroying the tooth-structure than by day. But I am inclined to think from my own observations that at night we have increased fermentation independent of any glands, and that this also tends to the destruction of the teeth. I am very certain, from clinical observation, that these are facts, for I have stopped erosion altogether by the use of an antacid.

Of course, the question is as yet much in the dark; I acknowledge that, and I suppose every one else does. The erosion by the old style of wire clasps, used many years ago, produced precisely the same effect as the fermentation ordinarily occurring at night, fermentation taking place between the clasp and the tooth.

Dr. Brubaker answered the preceding speakers in the following manner:

"I am well aware if you destroy the labial glands and diminish all secretion the lips will become very immobile; but, as a matter of fact, there are probably never more than a half-dozen of the glands which are diseased. The specimen shows there are fifty or sixty present, and the destruction of even a half-dozen would not interfere very materially with the quantity of secretion."

*Dr. Truman.*—How would you know when a gland is diseased? What are the indications?

*Dr. Brubaker.*—The indication of which gland is diseased is shown by pressing upon the gland. As Dr. Kirk demonstrated, pressure upon the glands forces out of it a fluid which is thin, watery, and very different from the normal secretion. And also the orifices of the glands, I am told, are nearly always enlarged, showing redness and congestion.

*Dr. Faught.*—I listened with great interest to the paper, and still I cannot entirely believe that the uric acid explanation is done away with. I base that upon the assertion within the paper. The paper lays particular emphasis upon the fact that this derangement in the gland may be due to some internal irritation within the gland itself. So it would seem to me that we are now touching only upon the process which takes place at one end of the line, and that it may still be possible to look for uric acid as an irritant producing this pathological condition.

*Dr. Gaskill.*—I think I agree quite fully with Dr. Brubaker so far as my limited knowledge of the subject will allow. He shows that the marked action in most cases of erosion must come from the glands of the lip, although the use of acids for systemic purposes may also have a marked effect on the teeth.

I have had several cases, and reported one which was very marked, of the use of the "microbe-killer." It was a lady who came to me several years ago, and when she first presented herself I noticed that some gold fillings in the front teeth looked as though they had never been properly dressed down. I asked her if she was taking acid medicines, and she said she was using considerable of the microbe-killer. She brought me some, and I found it was markedly acid in action. When I saw a similar indication in the teeth of other patients I questioned them, and I found three others using it. The result was the same with all. There was the greatest wasting away, both on the cutting-edge and the palatine walls, while the labial walls were less affected, owing to the protection given by the lip.

The subject was then passed and Incidents of Practice were taken up.

Dr. Fish then exhibited a black diamond, set in a bar of steel, that he claimed was most efficient in truing up old corundum wheels, a small tap, most valuable and inexpensive, and some asbestos rope that could be used as a rapidly adjusted investment for crowns or small pieces of bridge-work where soldering was required. Adjourned.

JOSEPH HEAD, M.D., D.D.S.,
*Editor Odontological Society of Pennsylvania.*

